# ThromboprophylaXIs with Factor XI/XIa inhibitors for venous thromboembolism

**DOI:** 10.1590/1677-5449.202300512

**Published:** 2023-06-19

**Authors:** Mateo Porres-Aguilar

**Affiliations:** 1 Texas Tech University Health Sciences Center El Paso, Paul L. Foster School of Medicine, Department of Internal Medicine, Divisions of Hospital and Adult Thrombosis Medicine, El Paso, Texas, United States of America.

Factor XI is the zymogen of XIa, a serine protease that is biosynthesized mainly in the liver and to a lesser extent in the pancreas and renal tubules. Factor XIa activates factors V, VIII, X, and XII and also inhibits important regulators such as tissue factor pathway inhibitors and von Willebrand factor-cleaving metalloproteinases.^1^ In vitro observations have shown that factor XI is converted to XIa by factor XII or thrombin or can even self-activate in the presence of polyanions (e.g. inorganic polyphosphates naturally present in thrombi), thereby playing a pivotal role in thrombin generation.^1^

The coagulation cascade has 3 major pathways: the contact pathway (intrinsic), the tissue factor pathway (extrinsic), and the common pathway. The contact pathway is activated when blood is exposed to an inflamed or damaged tissue surface. Factor XI/XIa plays an important role by mediating and modulating the amplification phase for the burst and growth/propagation of thrombi within the vessel. On the other hand, it plays only a minor stabilizing role in achieving hemostasis. Inhibition of XI/XIa could prevent pathologic thrombi that lead to venous thromboembolism (VTE), but still allow the tissue factor pathway to maintain functionality, minimizing the bleeding risks associated with other anticoagulants.^2^ Factor XI deficiency (Hemophilia-C/Rosenthal syndrome) found in Ashkenazi Jewish populations has been associated with lower cardiovascular events including VTE and ischemic stroke compared with the general population, and with a mild bleeding phenotype, and has been proven to be effective in preventing thrombosis without impairing overall hemostasis, resulting in identification of factor XI/XIa as an attractive therapeutic target for thromboprophylaxis in VTE.^2-4^

In a tireless effort to find *“safer and ideal”* anticoagulants, targeting a single serine protease preventing VTE while at the same time sparing hemostasis, researchers have paved the way for continuous innovation and development of new drugs that specifically target factor XI/XIa.^5^

As such, at least five new molecules have been developed and studied in early phase clinical studies: inhibition of the biosynthesis of factor XI/XIa in the liver with antisense oligonucleotides (ASOs), and direct factor XIa inhibition with monoclonal antibodies, small peptidomimetics, aptamers, and natural inhibitors.

The mechanism of action, pharmacodynamics, and pharmacokinetics of several factor XI/XIa inhibitors have been widely studied in phase-2 clinical trials. ASOs (e.g., IONIS-FXIRx, fesomersen, ISIS-FXIRx, and LICA) are administered subcutaneously in weekly doses and are highly bound to plasma proteins with limited glomerular filtration and urinary excretion.^2,6^ Monoclonal antibodies have emerged as therapeutic modalities in medicine over the past few decades; osocimab, abelacimab, xisomab 3G3, and BAY-1831865 bind directly to factor XI/XIa, resulting in faster onset of action. Their metabolism does not depend on the kidneys or on the liver and excretion is mostly via the reticuloendothelial system and phagocytic cells. They have half-lives of 30 to 44 days, allowing single intravenous (IV) doses to be used for VTE prophylaxis. ^2,6^ Small peptidomimetic drugs (e.g., milvexian, asundexian, and EP-7041 [frunexian]) directly inhibit factor XIa, are given either orally or IV in a daily or twice a day dosing regimen, and have hepatic (cytochrome P450) metabolism and limited renal excretion, with half-lives of 11 to 21 hours. ^2,6^ Natural peptides (e.g., fasxiator) inhibit activation of factors XI and XII, have so far been tested via IV, and have a very short half-life, although no studies in humans have so far been reported so far. DNA and RNA aptamers (e.g., FELIAP), are single-stranded oligonucleotides that recognize target proteins with high affinity and specificity; their onset of action is fast and administration is once daily, but unfortunately no human studies have been reported yet.^2^

Phase 2 clinical studies are currently emerging on factor XI/XIa inhibitors across a variety of clinical conditions, such as VTE prophylaxis in major orthopedic surgery patients, which is our focus for this editorial. However, phase 3 clinical trials are currently ongoing and planned for applications related to cardioembolic stroke prevention in non-valvular atrial fibrillation, post-acute coronary syndromes, post atherothrombotic stroke, end-stage renal disease, cancer-associated VTE, and peripheral arterial disease, to mention a few.

The *AXIOMATIC TKR* trial evaluated milvexian taken orally in 1,242 patients at 7 dosages (25, 50, or 200 mg daily or 25, 50, 100 and 200 mg twice daily) versus the low molecular weight heparin (LMWH) enoxaparin 40 mg subcutaneously daily, in patients with recent total knee replacement (TKA). Although randomization was open-label, patients that received milvexian at higher doses (100 mg twice daily or 200 mg twice daily) had decreases of 58% and 70% respectively in the risk of postoperative VTE compared to enoxaparin (RR of VTE:0.30; 95%CI: 0.15-0.62 for milvexian 200 mg twice daily; P < 0.001); bleeding of any severity occurred in 4% taking milvexian and in 4% taking enoxaparin; major bleeding (MB) or clinically relevant nonmajor bleeding (CRNMB) occurred in 1% and 2%, respectively, and the study concluded that milvexian was effective for VTE prevention in patients undergoing TKA and was associated with a low risk of bleeding overall.^7^

The *ANT-005 TKA* trial evaluated three IV doses of the postoperative monoclonal antibody abelacimab (30 mg, 75 mg, or 150 mg) against SQ enoxaparin in 412 patients undergoing TKA; the primary efficacy outcome was objective VTE; the principal safety outcome was a composite of MB or CRNMB up to 30 days postoperatively. VTE occurred in 13% in the 30-mg abelacimab group, 5% in the 75-mg abelacimab group, and 4% in the 150-mg abelacimab group, compared with 22% in the enoxaparin group. The 30-mg abelacimab regimen was noninferior to enoxaparin, and the 75-mg and 150-mg abelacimab regimens were superior to enoxaparin (P<0.001). Bleeding occurred in 2%, 2%, and none of the patients in the 30-mg, 75-mg, and 150-mg abelacimab groups, respectively, and in none of the patients in the enoxaparin group and the study concluded that a single IV dose of abelacimab after TKA was effective for VTE prevention and was associated with a low risk of bleeding.^8^

The *FOXTROT* trial was a randomized, open-label, adjudicator-blinded, phase 2 noninferiority trial of osocimab with observer blinding. A total of 813 patients undergoing TKA were randomized to receive one of the following: a single IV osocimab postoperative dose of 0.3 mg/kg (n = 107), 0.6 mg/kg (n = 65), 1.2 mg/kg (n = 108), or 1.8 mg/kg (n = 106); a preoperative dose of 0.3 mg/kg (n = 109) or 1.8 mg/kg (n = 108); 40 mg of SQ enoxaparin once daily (n = 105), or 2.5 mg of oral apixaban twice daily (n = 105). The primary outcome was VTE incidence up to 10 to 13 days postoperatively. The primary safety outcome of MB or CRNMB was assessed up to 10 to 13 days postoperatively. The preoperative dose of 1.8 mg/kg of osocimab met criteria for superiority compared with enoxaparin with a risk difference of 15.1% ( 11.3% vs 26.3%; P=0.007 for superiority); osocimab given postoperatively met criteria for noninferiority compared with enoxaparin at the 0.6-mg/kg dose, the 1.2-mg/kg dose, and the 1.8-mg/kg dose; MB or CRNMB was observed in up to 4.7% of those receiving osocimab, 5.9% receiving enoxaparin, and 2% receiving apixaban. The investigators concluded that further studies are needed to establish efficacy and safety of osocimab relative to standard VTE prophylaxis.^9^

The *FXI-ASO-TKA* study was an open-label, parallel-group study that randomly assigned 300 patients who were undergoing elective primary unilateral TKA to receive one of two SQ doses of FXI-ASO (200 mg or 300 mg), a second generation ASO, initiated 36 days before surgery, or 40 mg SQ enoxaparin once daily initiated the evening before or 6 to 8 hours after surgery. The primary efficacy outcome of objective VTE was noninferior to enoxaparin with the 200-mg regimen and superior with the 300-mg regimen (P<0.001). Bleeding occurred in 3% for both subgroups on FXI-ASO and in 8% of the patients on enoxaparin, but this difference did not reach statistical significance.^10^

Further confirmatory data of factor XI/XIa inhibitors efficacy and safety is highly anticipated in ongoing, large, phase 3, randomized, controlled clinical trials, to enable more robust conclusions to be drawn in regards net clinical benefit, and especially if any signs of harm may exist. It is not unreasonable that medical and surgical specialties, including vascular specialists, may incorporate frequent utilization of factor XI/XIa inhibitors into their daily clinical practice in the future. Unanswered questions remain, like regulatory approval, wide acceptance by clinicians and patients, and costs, especially if factor Xa and direct thrombin inhibitors become generic.
